# Pathophysiology and Prognostic Significance of Myoclonus in Hypoxic-Ischemic Encephalopathy

**DOI:** 10.1097/WNP.0000000000001272

**Published:** 2026-06-08

**Authors:** Markus Leitinger, Simone Beretta

**Affiliations:** * Department of Medicine and Surgery, University of Milano-Bicocca, Milan, Italy; and; † Department of Neurology, Fondazione IRCCS San Gerardo dei Tintori, Monza, Italy.

**Keywords:** Status epilepticus, Classification, Outcome

## Abstract

Cerebral hypoxia due to cardiac arrest is frequently complicated by myoclonus manifesting hours to days after the return of spontaneous circulation. However, myoclonus per se—without further characterization—lacks sufficient biological specificity and does not allow for reliable prognostic interpretation. Accumulating evidence underscores the critical role of EEG monitoring in the assessment of postanoxic patients, particularly with regard to background continuity and the presence of status epilepticus, as defined by the American Clinical Neurophysiology Society. These parameters have emerged as key determinants in prognostication. A structured research framework for the classification of postanoxic myoclonus has been proposed to mitigate the risk of false-positive predictions of poor neurological outcome and to prevent unwarranted withdrawal of life-sustaining therapy in selected patient subgroups. These subgroups are defined by continuous, nearly continuous, or discontinuous EEG background activity, particularly in the absence of concomitant status epilepticus. This framework constitutes a robust foundation for the systematic acquisition of relevant clinical and paraclinical data. In addition, a standardized communication tool may facilitate precise information exchange among physicians and efficient interprofessional patient management.

Following global cerebral hypoxia-ischemia, patients frequently develop abnormal motor activity within hours to days after the return of spontaneous circulation (ROSC) (Fig. 1). Clinically, myoclonus presents as a sudden, brief—lightninglike—involuntary muscle contraction (positive myoclonus) or as a transient inhibition of muscle contraction (negative myoclonus).^1–3^ These movements may occur as single or repetitive jerks, each typically lasting milliseconds. After cardiac arrest, myoclonus is observed in one-fifth to one-third of patients^4^ and may arise from cortical or subcortical structures, such as the brainstem or spinal cord, or have combined origins.^5,6^ Various body regions may be involved, and their distribution has been studied in relation to outcome.^7^ A poor neurological outcome was typically associated with bilateral synchronous (i.e., time locked right and left side) jerks that were generalized, axially distributed, or stereotyped in morphology.^8–10^ “Axial” referred to involvement of the face, eyes, jaw, neck, diaphragm, shoulders, and hips. “Stereotyped” describes jerks that are similar in distribution and appearance across episodes.^9^ By contrast, asynchronous, asymmetric, multifocal, or focal myoclonus—particularly when affecting distal extremities or varying in appearance—has been linked to better prognoses.^9,10^ Sedative-analgesic agents, neuromuscular blockade, and targeted temperature management (TTM) can delay or modify the presentation of postanoxic myoclonus introducing uncertainty regarding internal validity.^4,7^ Furthermore, little is known about the temporal evolution of myoclonus within individual patients, which may introduce a time-related sampling bias. The level of consciousness is a key prognostic factor. In awake patients, myoclonus usually manifests as action-induced and stimulus-triggered myoclonus termed Lance-Adams syndrome (LAS).^11^ However, in coma within 72 hours after ROSC, myoclonus that later evolves into LAS (early LAS, eLAS) may initially appear indistinguishable from myoclonus associated with poor outcome as in most cases.^12–14^

**FIG. 1.**
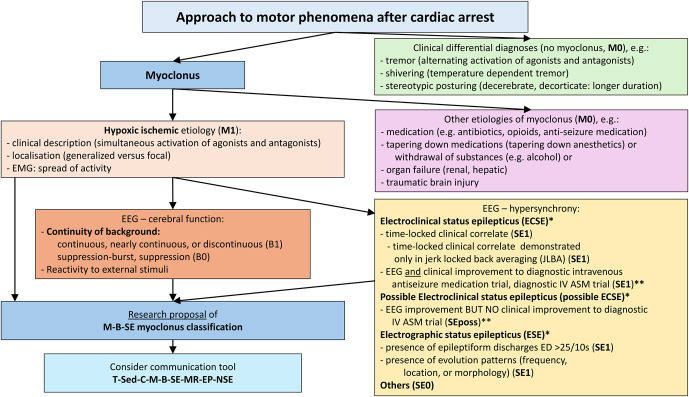
The approach to patients with myoclonus after cardiac arrest considering differential diagnoses of myoclonus, nonepileptic myoclonus, and a research proposal for the outcome relevant classification of posthypoxic myoclonus including EEG background and status epilepticus. *Definitions of status epilepticus (SE) referring to Salzburg criteria within the American Clinical Neurophysiology Society (ACNS)^15–18^: electrographic status epilepticus (ESE) in form of (1) epileptiform discharges (ED) greater than 2.5 Hz, or (2) evolution in frequency, location, or morphology; and electroclinical status epilepticus (ECSE) as (3) time-locked clinical correlate (i.e., jerks here); and (4) EEG and clinical improvement to diagnostic IV anti-seizure medication. Possible ECSE is a rhythmic or periodic pattern (RPP) that qualifies for the ictal-interictal-continuum (IIC) that shows EEG improvement with a parenteral antiseizure medication but without clinical improvement. All above mentioned kinds of status epilepticus need to last at least 10 continuous minutes or 12 cumulative minutes (i.e., ≥20%) of any 60-minute period of recording. **Proposal for EEG improvement and clinical improvement^19^: As an EEG response to IV ASM, there will be an EEG interval without IIC at least three times the longest prior spontaneous IIC-free EEG interval (if any), but lasting a minimum one continuous minute. In comatose patients, the background is not considered as part of a response to an IV ASM. In noncomatose patients, the following recommendations apply for determining a positive response of the EEG in IIC as defined (tailored to posthypoxic patients): If baseline EEG background frequency is known: Return to baseline (mild diffuse or focal slowing allowed) OR appearance of previously absent normal features (e.g., sleep spindles, posterior dominant rhythm). The clinical response to a diagnostic IV ASM trial can include improvement of consciousness or resolution of an ictal focal deficit and should be measured by using a standardized examination or scale, e.g., the NCSE response scale (NRS). In case that a clinical correlate, e.g., myoclonus, resolves but the EEG pattern does not respond, this is not considered successful treatment. ED, epileptiform discharges; NCSE, nonconvulsive status epilepticus.

Myoclonus must be differentiated from other motor phenomena frequently encountered in the intensive care unit. Tremor, the most common, features alternating activation of agonist and antagonist muscles across a joint, as seen in Parkinson disease or shivering.^20^ Stereotypic posturing, such as with decortication or decerebration, normally lasts seconds or even longer. Startle reactions are characterized by sudden involuntary movements provoked by alarm or pain stimuli.^21,22^

Additional etiologies of myoclonus include drug-induced myoclonus,^5,23–28^ withdrawal syndromes,^29^ or tapering of anesthetics such as propofol.^30^ Myoclonus may also occur secondary to renal or hepatic failure or traumatic brain injury.^31^

Electromyography can contribute to understanding pathophysiology: cortical myoclonus typically lasts <50 ms, whereas subcortical myoclonus is longer in duration. Patterns of distribution can also be detected appropriately.^32^ Sensitivity to stimuli is characteristic of cortical origin but can occasionally occur with subcortical, brainstem, or spinal generators.^5^

Distinguishing myoclonus from other postanoxic motor phenomena in comatose survivors after cardiac arrest remains a major diagnostic and prognostic challenge with crucial implications for outcome prediction and patient management.^13,33–35^ Accurate recognition of myoclonus is complicated by overlap in semiology and—most critically—heterogeneity in underlying EEG patterns.^13,34^ EEG remains essential for detecting hypersynchronous neuronal activity as in nonconvulsive status epilepticus (NCSE).

A time-locked coincidence between myoclonic jerks and mostly epileptiform discharges on EEG suggests a focal or multifocal cortical origin. Importantly, this time-locked clinical correlation of a motor phenomenon to an EEG feature (e.g., discharge or burst) fulfills the criterion of an electroclinical seizure and—when lasting ≥10 continuous minutes or ≥12 cumulative minutes (i.e., ≥20%) of any hour of recording—it constitutes electroclinical status epilepticus (ECSE) as defined by the Salzburg criteria of NCSE^15–17^ incorporated into the 2021 American Clinical Neurophysiology Society's Standardized Critical Care EEG Terminology.^15–18^ Jerked locked back averaging may reveal a cortical source even when discharges are not visually apparent. If muscle and movement artifacts obscure EEG interpretation, neuromuscular blockade should be considered to uncover underlying periodic or rhythmic discharges.

An EEG and clinical response to a diagnostic intravenous antiseizure medication trial (diagnostic IV ASM trial) represents the second form of ECSE.^18,19^ By contrast, “possible ECSE” is defined by rhythmic or periodic patterns on the ictal-interictal-continuum that improve electrographically—but not clinically—after a diagnostic IV ASM trial.^18,19^ The ictal-interictal-continuum includes periodic patterns, spike-and-wave/polyspike-and-wave/sharp-and-wave patterns, and lateralized rhythmic delta activity, each fulfilling additional criteria.^18^

Electrographic status epilepticus (SE) exists in two forms, i.e., (1) epileptiform discharges exceeding 25/10 seconds or (2) evolution in frequency, location, or morphology.^18^ All forms of NCSE need to fulfill the abovementioned criteria for duration. Cortical myoclonus may evolve into convulsive, i.e., bilateral tonic-clonic, seizures or status epilepticus (CSE) for which at least five continuous minutes duration apply.^18^ Subcortical lesions typically produce more generalized myoclonus without EEG correlates. Even so, favorable outcomes have been reported in both cortical and subcortical forms, with the latter appearing roughly half as often as cortical myoclonus.^34^

EEG also detects the integrity of generators of continuous background or, in turn, the appearance of suppression or suppression-burst patterns in case of widespread lesions. Thus, EEG is pivotal for individualized outcome estimation.

The biological and clinical significance of postanoxic myoclonus varies widely, reflecting heterogenous extent and neuroanatomical location of brain injury and respective generators of myoclonus in light of numerous substantial confounders. Comprehensive evaluation of overall and location-related cerebral damage should include neurological examination, laboratory parameters such as neuron-specific enolase (NSE), cerebral imaging in form of MRI, neurophysiological investigations of somatosensory evoked potentials (SEPs), and in particular EEG-investigation of cerebral functional performance and presence of NCSE.^4,36,37^ Ideally, prognostic interpretation should integrate all these parameters before drawing conclusions about myoclonus which puts an emphasis on terminology.

Historically, terms such as myoclonic status or status myoclonus have referred solely to the clinical persistence of jerks without characterization of injured brain structures, generators of myoclonus, and in particular EEG correlates. This approach inherently amalgamates heterogeneous clinical constellations and has thus been discouraged.^37^ The Babylonian confusion of tongues is accentuated by different definitions of myoclonic status, status myoclonus, myoclonic status epilepticus (MSE), myoclonus, early myoclonus, and late myoclonus across various studies identified in a recent systematic review on postanoxic myoclonus.^13^ To promote clarity, a structured notation system (M–B–SE framework) was proposed, condensing key clinical and electrophysiological features into a succinct communication tool^13^ (Table 1).

**TABLE 1.** Communication Tool for Standardized Reporting of Investigations in Patients After Cardiac Arrest Including the Research Proposal for Classification of Postanoxic Myoclonus (M-B-SE-classification Research Proposal)^13^

Domain	Abbreviation	Options	Example
Time since ROSC	T	hours (h), days (d)	T6h: 6 hours after ROSC
Sedation	Sed	1: Present	Sed1: Sedation, e.g., midazolam, still present as expected from context sensitive half-life or serum levels
		0: Absent	
		x: Unknown, not determined	
Coma	C	1: Present	C1: The patient cannot be aroused by noxious stimuli. C0: The patient is awake, somnolent, or stuporous.
		0: Absent	
		x: Unknown, not determined	
Myoclonus	M	1: Present	M1: The patient presents with myoclonus
		0: Absent	
EEG	E	x: Not performed yet	Ex: EEG not yet performed
EEG background	B	1: Continuous, nearly continuous, or discontinuous background	B0: EEG shows suppression or suppression burst background.
		0: Suppression-burst, or suppression.	
Status epilepticus	SE	1: Status epilepticus present*	SE1: One of the four criteria of NCSE are fulfilled* SEposs: The criteria for possible electroclinical SE are fulfilled*
		poss: possible electroclinical SE present	
		0: Criteria for SE not fulfilled	
Magnetic resonance imaging	MR	1: Lesions suspected to be caused by hypoxia are present	MR1: The MRI shows extensive restricted diffusion and reduced apparent diffusion coefficient (ADC)
		0: No lesions attributable to hypoxia	
		x: MRI not yet performed or findings unknown	
Evoked potentials	EP	1: Evoked potentials present	EP1: Evoked potential present on the left but missing on the right.
		0: Bilateral absent SEPs	
		x Eps not yet performed	
Neuron specific enolase	NSE	Level in µg/L	NSE18: The preliminary maximum level of NSE is 18 µg/L at the time after ROSC documented in T
		x: Not performed yet	
Example: T50h-Sed0-C1-M1-B1-SE0-MR1-EPx-NSE16: A patient investigated 50 hours after return of spontaneous circulation was still comatose despite being without sedation and showed myoclonus. The EEG revealed a continuous background and no status epilepticus. MRI presented mild hippocampal increase in diffusion weighted imaging and decrease in apparent diffusion coefficient. Evoked potentials have not been performed yet. Neuron specific enolase had a preliminary maximum of 16 µg/L.			

This communication tool was developed equally by De Stefano and Leitinger.^13^ Continuous: <1% periods of suppression (<10 µV) or attenuation (≥10 µV but <50% of background voltage); Nearly continuous: 1% to 9% periods of suppression attenuation; Discontinuous: 10% to 49% periods of suppression or attenuation; Burst-suppression or Burst-attenuation: 50% to 99% periods of suppression or attenuation; Suppression: >99% periods of suppression or attenuation.^18^ ROSC, return of spontaneous circulation

* Definitions of status epilepticus (SE) referring to Salzburg criteria within the American Clinical Neurophysiology Society (ACNS)^15–18^: electrographic status epilepticus (ESE) in form of (1) epileptiform discharges greater than 2.5 Hz, or (2) evolution in frequency, location, or morphology; and electroclinical status epilepticus (ECSE) as (3) time-locked clinical correlate (i.e., jerks here); and (4) EEG and clinical improvement to diagnostic IV anti-seizure medication. Possible ECSE is a rhythmic or periodic pattern (RPP) that qualifies for the ictal-interictal-continuum (IIC) that shows EEG improvement with a parenteral antiseizure medication BUT without clinical improvement. All above mentioned kinds of status epilepticus need to last at least 10 continuous minutes or 12 cumulative minutes (i.e., ≥20%) of any 60-minute period of recording.

In the case of myoclonus (M1), the presence of (1) a continuous, nearly continuous, or discontinuous background (each B1), OR (2) suppression, or suppression-burst (each B0) can be documented along with the presence of status epilepticus (SE1) or its absence (SE0) (Fig. 2), an EEG not yet performed (Ex), and several other relevant parameters, for example, MR1 if hypoxic signal alterations were demonstrated, or EP1 for present evoked potentials.

**FIG. 2.**
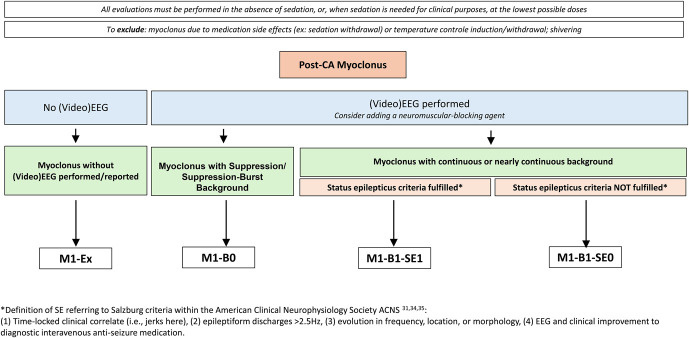
Research proposal on terminology based on EEG correlate of myoclonus (M1). Note of caution: this classification needs future validation by an international expert panel and integration into a multimodal concept of outcome estimation. *Definitions of status epilepticus (SE1) referring to Salzburg criteria within the American Clinical Neurophysiology Society (ACNS)^15–18^: electrographic status epilepticus (ESE) in form of (1) epileptiform discharges greater than 2.5 Hz, or (2) evolution in frequency, location, or morphology; and electroclinical status epilepticus (ECSE) as (3) time-locked clinical correlate (i.e., jerks here); and (4) EEG and clinical improvement to diagnostic IV anti-seizure medication. Possible ECSE (SEposs) is a rhythmic or periodic pattern (RPP) that qualifies for the ictal-interictal-continuum (IIC) that shows EEG improvement with a parenteral antiseizure medication but without clinical improvement. All above mentioned kinds of status epilepticus need to last at least 10 continuous minutes or 12 cumulative minutes (i.e., ≥20%) of any 60-minute period of recording). 0, absent; 1, present; B, background; CA, cardiac arrest; M1-B0, myoclonus associated with suppression/suppression burst background EEG; M1-B1-SE0, myoclonus with nonsuppression/suppression burst background without status epilepticus EEG; M1-B1-SE1, myoclonus with nonsuppression/suppression burst background and status epilepticus-electroencephalogram (Reproduced with permission from^13^ [De Stefano & Leitinger et al 2025]).

For instance, a patient designated T40 h–Sed0–C1–M1–B1–SE0–MRx–EPx–NSE18 would have, 40 hours post-ROSC (T40h), no sedation (Sed0), in coma (C1), presence of myoclonus (M1), continuous EEG background (B1) without SE (SE0), a preliminary maximum NSE 18 µg/L, with MRI and SEPs pending (MRx-EPx).

Conversely, a case labeled T6d–Sed0–C1–M1–B0–SE0–MR1–EP0–N90 indicates day 6 post-ROSC, no sedation, coma with myoclonus, burst-suppression EEG, no SE, MRI-detected lesions, absent SEPs, and a maximum NSE of 90 µg/L. This framework allows clinicians to rapidly grasp the patient's state in a busy intensive care unit and facilitates stratification for inclusion into dedicated studies or comparisons across heterogeneous studies.

## PATHOPHYSIOLOGICAL MECHANISMS

Cerebral ischemia and hypoxia are prototypical causes of neuronal injury. Insights from these mechanisms inform our understanding of the clinical and electrophysiological presentations of other etiologies.

Cortical neurons largely consist of two populations: excitatory glutamatergic projection neurons (pyramidal neurons), which show many dendritic spines and long-range axons, and inhibitory GABAergic interneurons (GaINs), which demonstrate few or no dendritic spines and form local connections.^38^ Together, they maintain network stability and enable precise cortical function.^39^ GaINs constitute roughly 30% of all neocortical and approximately 15% of all hippocampal neurons.^40,41^ At the intracellular level, the balance of intracellular calcium is maintained by calcium-binding proteins employing EF-hand domains (specific loop-helix-loop motives in their tertiary structure) to buffer Ca^2+^ influx.^42^

Excessive metabolic demand due to the generation of gamma oscillations, reactive oxygen species generation, and susceptibility to calcium overload render GaINs particularly vulnerable to oxygen/glucose deprivation accentuated by mitochondrial complex I/IV dysfunction.^43^ Calcium-binding proteins provide transient protection but become depleted under sustained stress.^38^

Protracted hypoxia challenges both cell types; however, tolerability of prolonged cerebral ischemia is substantially reduced in GaINs; neuronal cell injury and cell death are mediated by the neurotoxicity and excitotoxicity of glutamate and increased intracellular concentrations of Ca^2+^.^38^ The periodic EEG patterns found in patients with cerebral hypoxia might reflect disinhibition of cortical pyramidal neurons, as suggested by computational models.^44^

As a note of caution, most evidence available is derived from animal research *in vivo* or *in vitro*.^41^ The results found at the correlation level need further evaluation by knock-out models or overexpression approaches. Furthermore, the complex interactions of intraneuronal processes, including mitochondrial responses and changes in the plasma membrane, need to be matched with interneuronal and neuronal–glial interactions alongside with angiological and immunological alterations, which are challenging to address in most models. As a further limitation, when referring to results with a particular cell type, for example, GABA-ergic neurons, one has to consider the large number of different cell groups within this population.^40^

Future research should use specific investigation of GaINs, e.g., by special radioactive tracers for imaging by single photon emission computer tomography or positron emission computed tomography.^45^ In addition, pharmacological approaches should provide protection or curation either selective for GaINs or for all involved cell types as the current emphasis relies on inhibition of overly active glutamatergic neurons.^46^

## PROGNOSTIC SIGNIFICANCE OF POSTANOXIC MYOCLONUS AND ITS RELATIONSHIP WITH EEG PATTERNS

Postanoxic myoclonus in comatose patients after cardiac arrest still represents one of the most challenging entities for prognostication and management in neurocritical care.^34,47,48^ Its multifaceted clinical and neurophysiological presentations transcend traditional binary “benign” versus “malignant” prognostic markers.^49,50^ Historically, certain subforms of myoclonus observed within 72 hours postarrest—including generalized myoclonus, status myoclonus/myoclonic status, and MSE—were primarily viewed as markers of devastating brain injury, leading many clinical guidelines to equate their presence with poor prognosis and support consideration of withdrawal of life-sustaining therapy.^51,52^ Contemporary data challenge this view. Pooled analyses and systematic reviews have shown that outcome prediction based solely on clinical myoclonus is unreliable; instead, EEG background continuity and presence of SE are decisive prognostic variables.^13,49^

A recent comprehensive systematic review of 119 studies and nearly 4,000 patients^13^ stratified the outcomes after cardiac arrest by different definitions of myoclonus, EEG background, and presence of SE. Clinical definitions alone, such as “myoclonus,” “status myoclonus/myoclonic status,” or “MSE” demonstrated strikingly low rates of favorable long-term neurological outcome (cerebral performance category [CPC] 1 to 2, indicating independence in daily life activities) of 9.8% for myoclonus, 5.8% for status myoclonus/myoclonic status, and 5.7% for MSE (Table 2). In contrast, the eLAS yielded 82% good outcomes, underscoring the importance of the electrophysiological context. The pivotal finding was that stratification by EEG background provided a far more discriminative model of neurological recovery than clinical definitions alone. Among patients with postcardiac arrest myoclonus, the outcomes varied substantially according to the EEG background. Those with myoclonus and suppression, suppression-burst, or unreactive background (SB/U) had a good neurological outcome (CPC 1–2) in only 1.6% of the cases. By contrast, 22.2% of patients with myoclonus and a non-SB/U background, i.e., continuous, nearly continuous, and discontinuous background—without SE achieved good outcomes. Interestingly, the subgroup with myoclonus, non-SB/U background, and concomitant SE showed an intermediate rate of good outcome (11.2%).

**TABLE 2.** Distribution of Good Neurologic Outcomes (CPC 1–2) in Patients With Myoclonus Stratified Both by Clinical Definition and Underlying EEG Patterns, From Patients With Reported Outcomes (N) in a Recent Systematic Review^13^

Patient Group	Definition, Characteristic Features	N	Good Outcome (CPC 1–2) (%)
Myoclonus (any type)	Variable definitions	2008	9.8
Status myoclonus/myoclonic status	Variable definitions	487	5.8
Myoclonic status epilepticus	Variable definitions	437	5.7
Early Lance-Adams syndrome	Variable definitions	33	81.8
Myoclonus + SB/U background	EEG: SB/U background, with or without status epilepticus (SE)	577	1.6
Myoclonus + non-SB/U + SE	EEG: Continuous, nearly continuous, discontinuous, or reactive background with SE	391	11.2
Myoclonus + non-SB/U, no SE	EEG: Continuous, nearly continuous, discontinuous, or reactive background without SE	404	22.2

This table highlights the prognostic value of integrating EEG correlates: background continuity (continuous, nearly continuous, and discontinuous vs. suppressed, suppression-burst, or unreactive) and status epilepticus (presence vs. absence) can substantially alter outcome expectations for patients who otherwise share the clinical presentation of myoclonus.

N, number of patients with reported outcomes. For details see also explanations in Table 1; SB/U, suppression-burst or unreactive.

These results parallel findings from a recent pooled analysis of the TELSTAR trial and two observational registries,^53^ which further emphasized the role of EEG background and multimodal prognostic indicators in clinical outcomes after postanoxic SE. Among 274 patients with postanoxic definite or possible SE,^54^ approximately 50% exhibited motor manifestations. A good neurological outcome (CPC 1–2 at 3 months) occurred in only 3.2% of those patients with a discontinuous or suppression-burst EEG background before SE onset; by contrast, patients whose SE emerged from a continuous or nearly continuous background had a 22.2% chance of recovery. This increased to 25% in the absence of 2 or more poor prognostic indicators (brainstem reflexes, NSE, neuroimaging, evoked potentials, suppression or burst suppression EEG background, myoclonus occurring ≤72 h) as stated in the ERC/ESICM guidelines.^51^ Notably, higher discharge frequency (>2.5 Hz), cessation of SE, and higher doses of antiseizure medications (levetiracetam and valproic acid) were associated with better outcomes in patients with SE and favorable prognostic indicators; however, these associations faded in the context of possible SE or suppression or suppression burst EEG backgrounds.

Similarly, a previous prospective cohort study by Beretta et al.^55^ explicitly pursued an intensive and prolonged standardized treatment protocol in patients with refractory postanoxic SE guided by continuous EEG monitoring and multimodal prognostic indicators, that is, patients were treated as long as no unfavorable indicators were present. Survival and good neurologic outcomes at 6 months reached 54% and 44%, respectively, in the refractory SE group, a striking contrast to near-zero outcomes in patients with generalized periodic discharges (GPDs) or high-risk nonepileptiform backgrounds. In this study, nonconvulsive SE was the most common type (55.6%), while motor manifestations were observed in 44.4% and 18.7% of the cases, respectively. Importantly, early EEG background reactivity was strongly associated with neurological recovery, again reinforcing that the EEG context supersedes clinical semiology in prognostication.

Thus, the practical implications for neurocritical care are substantial. First, the data suggest that multimodal prognostication (incorporating EEG background, SE criteria, and well-established clinical and paraclinical markers) is mandatory for optimal outcome prediction (Table 2).^4,13,50^ Second, aggressive antiseizure therapy may be justified in patients with postanoxic SE (either convulsive or nonconvulsive) on a continuous background in EEG, particularly when poor multimodal prognostic indicators are absent. Conversely, the emergence of myoclonus in the setting of a suppressed or SB/U background, particularly when accompanied by concordant multimodal prognostic indicators of severe anoxic brain injury, is associated with a very poor prognosis, with meaningful recovery highly unlikely despite therapy. As a note of caution, highly frequent discharges may impair the detection of background, especially if slow waves follow spikes or sharp waves; here, the reduction of epileptiform activity is a prerequisite for the determination of the EEG background.

These advances have attempted to overcome the significant limitations of previous literature in the form of publication bias, self-fulfilling prognostication where withdrawal of life-sustaining therapy is enacted on isolated markers and inconsistent EEG reporting. Studies often lack granular descriptions of myoclonus semiology and rarely provide EEG data associated with myoclonus. Furthermore, the influence of sedation, hypothermia, and antiseizure medications on both EEG patterns and clinical outcomes must be considered when interpreting available evidence. Nonetheless, the convergent findings across large cohorts, prospective studies, and systematic reviews strongly advocate for an integrated EEG-based approach, moving beyond the era of “myoclonus inevitably means poor outcome” to a nuanced, multimodal paradigm.^53,56^

## CONCLUSIONS

The prognostic significance of postanoxic myoclonus should be interpreted in the context of the underlying EEG pattern, particularly background continuity and the presence of SE, as outlined in the research proposal for the M-B-SE classification framework. Myoclonus occurring on a continuous background and without SE should be regarded with cautious optimism, given a potential for favorable outcome in more than 20% of cases, provided additional adverse multimodal prognostic indicators are absent. When myoclonus is accompanied by a continuous background and SE, the likelihood of a good outcome might be enhanced by intensive SE-directed treatment, while the current evidence does not establish a clear causal treatment benefit. By contrast, myoclonus emerging from a suppressed or suppression–burst background, with or without concomitant SE, remains strongly associated with poor recovery. Future studies should prioritize terminological standardization and further evaluation of EEG-focused categorization schemes to optimize outcome prediction and guide ethically informed decisions regarding treatment intensity and withdrawal of life supporting care in comatose survivors of cardiac arrest.
